# Extremely Preterm Infant Admissions Within the SafeBoosC-III Consortium During the COVID-19 Lockdown

**DOI:** 10.3389/fped.2021.647880

**Published:** 2021-07-12

**Authors:** Marie Isabel Rasmussen, Mathias Lühr Hansen, Gerhard Pichler, Eugene Dempsey, Adelina Pellicer, Afif EL-Khuffash, Shashidhar A, Salvador Piris-Borregas, Miguel Alsina, Merih Cetinkaya, Lina Chalak, Hilal Özkan, Mariana Baserga, Jan Sirc, Hans Fuchs, Ebru Ergenekon, Luis Arruza, Amit Mathur, Martin Stocker, Olalla Otero Vaccarello, Tomasz Szczapa, Kosmas Sarafidis, Barbara Królak-Olejnik, Asli Memisoglu, Hallvard Reigstad, Elżbieta Rafińska-Ważny, Eleftheria Hatzidaki, Zhang Peng, Despoina Gkentzi, Renaud Viellevoye, Julie De Buyst, Emmanuele Mastretta, Ping Wang, Gitte Holst Hahn, Lars Bender, Luc Cornette, Jakub Tkaczyk, Ruth del Rio, Monica Fumagalli, Evangelia Papathoma, Maria Wilinska, Gunnar Naulaers, Iwona Sadowska-Krawczenko, Chantal Lecart, María Luz Couce, Siv Fredly, Anne Marie Heuchan, Tanja Karen, Gorm Greisen

**Affiliations:** ^1^Department of Neonatology, Rigshospitalet, Copenhagen, Denmark; ^2^Department of Pediatrics, Medical University of Graz, Graz, Austria; ^3^Infant Centre and Department of Paediatrics and Child Health, University College Cork, Cork, Ireland; ^4^Department of Neonatology, La Paz University Hospital, Madrid, Spain; ^5^Department of Pediatrics, The Royal College of Surgeons in Ireland, Dublin, Ireland; ^6^St. Johns Medical College Hospital, Bengaluru, India; ^7^Department of Neonatology, 12 Octubre University Hospital, Madrid, Spain; ^8^Neonatology Department, Hospital Clínic-Maternintat, Barcelona, Spain; ^9^Department of Neonatology, Kanuni Sultan Suleyman Training and Research Hospital, Istanbul, Turkey; ^10^Division of Pediatrics - Neonatal-Perinatal, University of Texas (UT) Southwestern, Dallas, TX, United States; ^11^Division of Neonatology, Department of Pediatrics, Uludag University Medical Faculty, Uludag, Turkey; ^12^Division of Neonatology, Department of Pediatrics, University of Utah, Salt Lake City, UT, United States; ^13^Third Faculty of Medicine, Institute for the Care of the Mother and Child, Charles University, Prague, Czechia; ^14^Center for Pediatrics, Department of Neonatology, Medical Center, University of Freiburg, Freiburg, Germany; ^15^Department of Neonatology, Gazi University Hospital, Ankara, Turkey; ^16^Division of Neonatology, Instituto del Niño y del Adolescente, Hospital Clinico San Carlos-Health Research Institute San Carlos (IdISSC), Madrid, Spain; ^17^Department of Neonatal-Perinatal Medicine, Saint Louis University School of Medicine, St. Louis, MO, United States; ^18^Neonatal and Pediatric Intensive Care Unit, Children's Hospital Lucerne, Lucerne, Switzerland; ^19^Department of Neonatology, Hospital Universitario de Tarragona Juan XXIII, Tarragona, Spain; ^20^Department of Neonatology, Neonatal Biophysical Monitoring and Cardiopulmonary Therapies Research Unit, Poznan University of Medical Sciences, Poznań, Poland; ^21^First Department of Neonatology, Aristotle University, Hippokrateion General Hospital, Thessaloniki, Greece; ^22^Department of Neonatology, Wrocław Medical University, Wrocław, Poland; ^23^Department of Neonatology, Marmara University Pendik Training and Research Hospital, Istanbul, Turkey; ^24^Department of Neonatology, Haukeland University Hospital, Bergen, Norway; ^25^Department of Neonatology, Centrum Medyczne “Ujastek”, Krakow, Poland; ^26^Department of Neonatology and Neonatal Intensive Care Unit (NICU), University Hospital of Heraklion, Crete, Greece; ^27^Department of Neonatology, Children's Hospital of Fudan University, Shanghai, China; ^28^Neonatal Intensive Care Unit (NICU), Department of Pediatrics, Patras Medical School, Patras, Greece; ^29^Neonatal Intensive Care Unit, Department of Pediatrics, University of Liege, Liege, Belgium; ^30^Neonatal Intensive Care Unit (NICU), Tivoli Hospital, La Louviere, Belgium; ^31^S.C. Neonatologia - Pres Osp S. Anna – Citta della Salute e della Scienza di Torino, Torino, Italy; ^32^Department of Neonatology, Guangzhou Women and Children's Medical Center, Guangzhou, China; ^33^Department of Neonatology, Aalborg University Hospital, Aalborg, Denmark; ^34^Department of Neonatology, AZ St-Jan Bruges, Bruges, Belgium; ^35^Department of Neonatology, University Hospital Motol, Prague, Czechia; ^36^Department of Neonatology, Hospital Sant Joan de Déu, Barcelona, Spain; ^37^Department of Neonatology, Fondazione IRCCS Ca' Granda Ospedale Maggiore Policlinico Milan, Milan, Italy; ^38^Department of Clinical Sciences and Community Health, University of Milan, Milan, Italy; ^39^Neonatal Intensive Care Unit, “Alexandra” University and State Maternity Hospital, Athens, Greece; ^40^Neonatology Department, Centre of Postgraduate Medical Education, Warsaw, Poland; ^41^Department of Neonatology, University Hospital Leuven, Leuven, Belgium; ^42^Department of Neonatology, Collegium Medicum in Bydgoszcz Nicolaus Copernicus University, Toruń, Poland; ^43^Department of Neonatology, Grand Hôpital de Charleroi (GHdC), Charleroi, Belgium; ^44^Neonatology Department, University Clinical Hospital of Santiago de Compostela, Health Research Institute of Santiago de Compostela, Santiago, Spain; ^45^Department of Neonatology, Oslo University Hospital, Oslo, Norway; ^46^Department of Neonatology, Royal Hospital for Children, Glasgow, United Kingdom; ^47^Department of Neonatology, University Hospital Zurich, Zurich, Switzerland

**Keywords:** extremely preterm, COVID-19, randomized clinical trial, pandemic, observational study, neonatal intensive care unit admission

## Abstract

**Objective:** To evaluate if the number of admitted extremely preterm (EP) infants (born before 28 weeks of gestational age) differed in the neonatal intensive care units (NICUs) of the SafeBoosC-III consortium during the global lockdown when compared to the corresponding time period in 2019.

**Design:** This is a retrospective, observational study. Forty-six out of 79 NICUs (58%) from 17 countries participated. Principal investigators were asked to report the following information: (1) Total number of EP infant admissions to their NICU in the 3 months where the lockdown restrictions were most rigorous during the first phase of the COVID-19 pandemic, (2) Similar EP infant admissions in the corresponding 3 months of 2019, (3) the level of local restrictions during the lockdown period, and (4) the local impact of the COVID-19 lockdown on the everyday life of a pregnant woman.

**Results:** The number of EP infant admissions during the first wave of the COVID-19 pandemic was 428 compared to 457 in the corresponding 3 months in 2019 (−6.6%, 95% CI −18.2 to +7.1%, *p* = 0.33). There were no statistically significant differences within individual geographic regions and no significant association between the level of lockdown restrictions and difference in the number of EP infant admissions. A *post-hoc* analysis based on data from the 46 NICUs found a decrease of 10.3%in the total number of NICU admissions (*n* = 7,499 in 2020 vs. *n* = 8,362 in 2019).

**Conclusion:** This *ad hoc* study did not confirm previous reports of a major reduction in the number of extremely pretermbirths during the first phase of the COVID-19 pandemic.

**Clinical Trial Registration:** ClinicalTrial.gov, identifier: NCT04527601 (registered August 26, 2020), https://clinicaltrials.gov/ct2/show/NCT04527601.

## Introduction

On the 11th of March 2020, COVID-19 was declared a pandemic by the World Health Organization, which led to an almost worldwide lockdown (1). Major reductions in the birth rates of extremely preterm and extremely low birth weight infants during the lockdown have been reported in Danish (2) and Irish (3) studies. The Danish study reported a decrease in the number of infants born extremely preterm (EP, infant born before 28 weeks gestational age); one EP infant from the 12th of March to the 14th of April of 2020 compared to a mean of 11.4 over the same time period of the preceding 5 years, in all of Denmark (2). The same trend was seen in the Irish study, relying on data from one hospital in Ireland, where no extremely low birth weight infants (<1,000 gm) were born from January to April in 2020, compared to a mean of 4.9 from January to April during the preceding 20 years (3). A Dutch study utilizing a 10-year time period found evidence of a moderate reduction of the whole spectrum of pre-maturity, follow the implementation of COVID-19 mitigation measures (4). In contrast, a Nepalese study (5) based on nine health institutions reported an increase in the preterm birth rate from 16.7 to 20.0%. A birth cohort from two hospitals in Philadelphia, United States, found no significant difference in preterm birth rates, when comparing the period of March to June 2020 (283 preterm births), to the same months during 2018 and 2019 (617 preterm births in total).

The total number of extremely preterm and extremely low birth weight infants in these studies are small and thus, the results should be interpreted with caution (6). The case was taken up in the New York Times, which reported that several neonatologists from neonatal intensive care units (NICU) worldwide, had observed a decrease in local pre-maturity rates, while other neonatologists observed the contrary (7).

**Figure 1:**
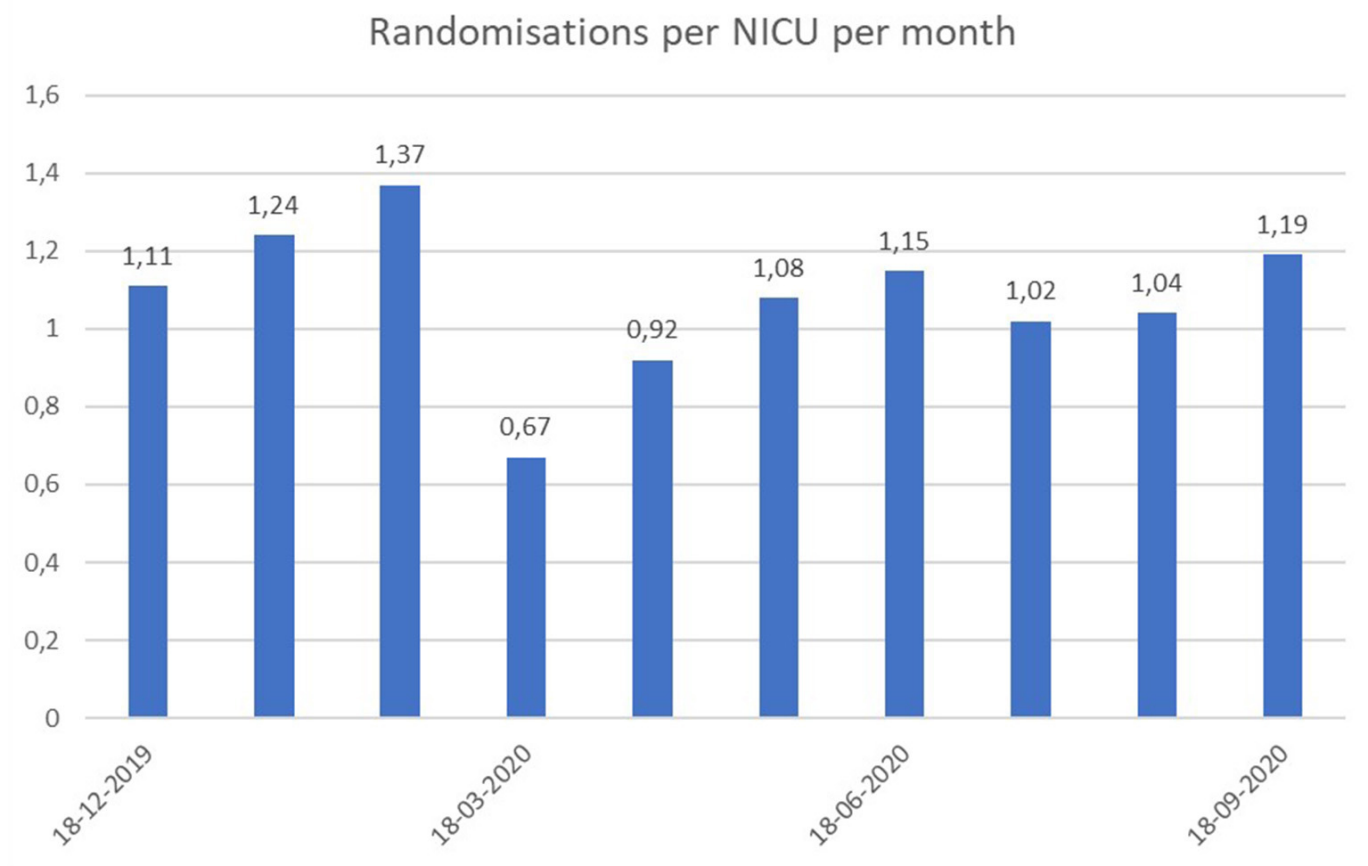
Number of randomisations per month within each NICU actively randomizing infants in the SafeBoosC-III trial during the last 9 months.

The SafeBoosC-III trial investigates the effects of treatment guided by cerebral near-infrared spectroscopy monitoring in extremely preterm infants (8). Despite having a vulnerable population, potentially with an increased risk of complications to a COVID-19 infection, the trial was able to proceed in most countries. Furthermore, several NICUs were opened for randomisation in this time period. However, the average monthly number of randomisations per NICU was almost halved in March, simultaneously with the spread of COVID-19 across Europe (Figure 1). Given the contradictory reports in the published studies on pre-maturity rates during the COVID-19 lockdown, and the variability in observations from neonatologists worldwide and within the SafeBoosC consortium, we decided to formally investigate the effect of the COVID-19 pandemic within our NICUs. For the SafeBoosC-III trial, the difference in EP admissions is most relevant, as this is the eligibility criteria.

The purpose of this study was to evaluate, if the number of admitted EP infants differed in NICUs within the SafeBoosC-III consortium during the global lockdown. Furthermore, being an international consortium, we wished to evaluate if there were differences within geographical regions, or associations between the level of local lockdown restrictions and difference in the number of EP infant admissions.

## Methods and Study Design

This is a retrospective, observational study, based on the NICUs in the consortium of the SafeBoosC-III randomized clinical trial (8). The principal investigators from all of the 79 NICUs in the consortium, were invited to participate in this study by e-mail and asked to report the following information: (1) the number of EP infants admitted to their NICU within the 3 months, where the lockdown restrictions were most rigorous during the first phase of the COVID-19 pandemic, (2) the number of EP infants admitted within the corresponding 3 months of 2019, and (3) the level of restrictions imposed upon the public, during the most rigorous 3 months of the lockdown period, in a Likert scale format from one to five. The consecutive 3 months where the lockdown restrictions were most rigorous during the first phase of the COVID-19 pandemic, were subjectively defined by local principal investigators. The scale used to classify the level of lockdown restriction, is a modified scale inspired from the New Zealand COVID-19 alert system (9), with one being the normal state of society, two being mild restrictions, three being moderate restrictions, four being strong restrictions and five being very strong restrictions (full scale in Appendix 1). Principal investigators reported that the data on the number of EP infant admissions was collected from admissions logbooks, NICU databases, hospital databases, and in one case, from a national registry.

Investigators were also asked to categorize the impact of the COVID-19 lockdown on the everyday life of a pregnant woman, and additionally, if they believed that the lockdown restrictions could possibly lead to a non-admittance of EP infants (e.g., intrauterine death due to delayed admittance of the mother, no possibility to transfer the baby from place of birth to a tertiary centre). Lastly, investigators were asked if there within the last year had been any major changes in the organization of perinatal care in their area/region, which may have changed the number of EP infant admissions to their respective NICU. The full data report template and data set can be found in Appendices 1, 2.

### Outcomes

The primary outcome was the difference in the total number of EP infant admissions during the 3 months with the most rigorous lockdown restrictions during the first phase of the COVID-19 pandemic, compared to the corresponding 3 months in 2019.

Secondary outcomes were

1. The difference in the number of EP infant admissions within the following regions: Asia, Eastern Europe, Southern Europe, Northern Europe, Western Europe, North America
2. The correlation between the level of the local lockdown restrictions and difference in the number of EP infant admissions.

Exploratory outcomes were (1) the likelihood that restrictions inside or outside health institutions in the investigators country/region, have led to non-admittance of EP infants, (2) if any major changes in the organization of perinatal care had occurred locally, which may have changed the number of admissions of EP infants, and (3) the impact of the COVID-19 lockdown on the everyday life of a pregnant woman.

### Statistical Analysis

The statistical analysis was decided a priori. The total number of EP infant admissions, during the 3 months with the most rigorous lockdown restrictions in 2020, during the corresponding three months in 2019, within each region and within each level of lockdown restriction, were to be reported as numbers (*n*). The primary outcome, as well as the secondary outcome regarding the difference in the number of EP infant admissions within each geographical region, were analyzed using Chi-square tests for 1 × 2 tables. To analyse the correlation between the local level of lockdown restrictions and the difference in number of EP infant admissions, we used simple linear regression. The exploratory outcomes did not undergo statistical analysis but were reported and discussed. For the primary outcome, an alfa level of 5% was chosen as a threshold for significance. To correct for multiple testing in the secondary outcomes, we chose an alfa level of 1%. Statistics were conducted in IBM SPSS Statistics 25 (IBM, Armonk, NY, US).

In a previous funding application for the SafeBoosC-III trial, the 93 NICUs taking part in the application, had reported an average of 30 admissions per year, i.e., 7 per 3 months period. Therefore, if half of the NICUs in the SafeBoosC-III consortium (i.e., 40 NICUs) participated, we would expect a total of 280 EP infant admissions within the 40 NICUs in 2019. Thus, a 16.5% difference in the primary outcome would be needed, to show a statistical significance with a 5% alfa level as threshold.

Pre-defined regions based on the active SafeBoosC-III NICUs can be found in Table 1.

**Table 1:** Pre-defined regions within the 79 NICUs in the SafeBoosC-III consortium.

**Asia**	**Eastern Europe**	**Southern Europe**	**Northern Europe**	**Western Europe**	**North America**
India, China	Austria, Poland, Czech Republic, Ukraine, Turkey	Spain, Portugal, Italy, Greece	Denmark, Norway	Germany, UK, Ireland, Switzerland, Belgium	USA

### *Post-hoc* Data Collection and Analysis

During the review process of a previous submission for publication of this manuscript, we were asked to provide the total number of NICU admissions. Therefore, in a second round, investigators were asked to report the total number of infant admissions to their NICU within the 3 months where the lockdown restrictions were most rigorous during the first phase of the COVID-19 pandemic, as well as within the corresponding 3 months of 2019.

### Ethical Considerations

The Danish National Committee on Health Research Ethics ruled that this study did not constitute a health research project. Therefore, permission to conduct this study was not necessary. To our knowledge, two participating European NICUs consulted with their local ethical committee, regarding the need for approval, but this was not required either. This study was registered on clinicaltrials.gov (NCT04527601) before any data collection started.

## Results

**Figure 2:**
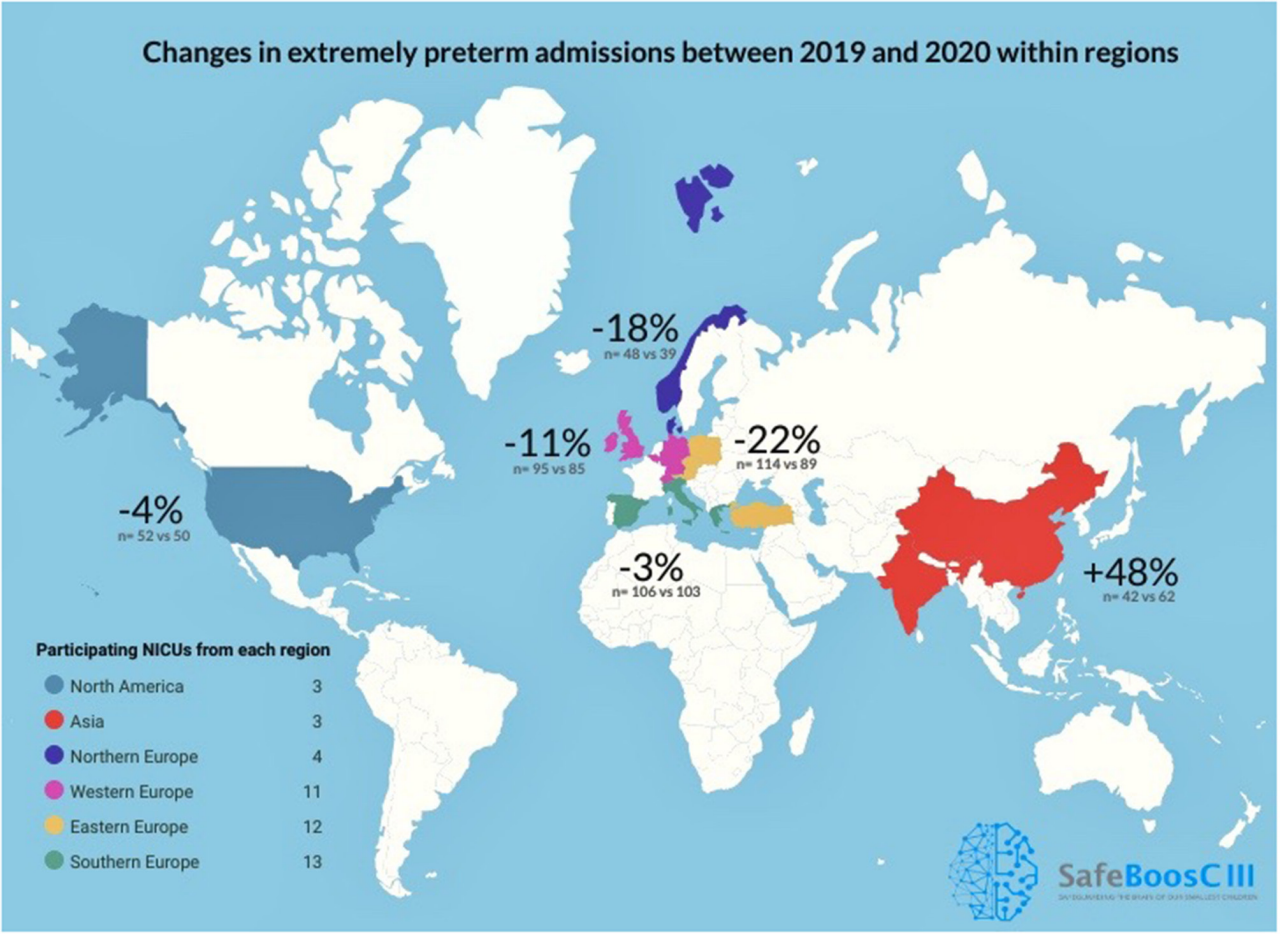
Change in percent in extremely preterm admissions between the 3 months with the most rigorous lockdown restrictions in 2020, compared to the corresponding months of 2019 in the 46 participating NICUs in the SafeBoosC-III consortium.

Data was delivered from 46 NICUs on a total of 885 EP infants across 17 countries (Austria, Belgium, China, Czech Republic, Denmark, Germany, Greece, India, Ireland, Italy, Norway, Poland, Switzerland, Spain, Turkey, UK, USA). The data source was reported to be an admission logbook (*n* = 16), a NICU database (*n* = 19), a hospital database (*n* = 10), and a national database (*n* = 1). During the 3 months with the most rigorous lockdown restrictions in 2020 the number of EP infant admissions was 428, compared to 457 in the corresponding 3 months of 2019 (a decrease of 6.6%, 95% CI −18.2 to +7.1%, *p* = 0.33). No significant differences were found in the number of EP admissions between 2020 and 2019 within the individual six pre-defined geographical regions (Table 2 and Figure 2).

**Table 2:** EP infant admissions in 2020 and 2019 within each pre-defined geographic region.

**Region**	**EP infant admissions during 3 months of most rigorous restrictions in 2020 (*n*)**	**EP infant admissions during the corresponding 3 months of 2019 (*n*)**	***p*-value**
North America	50	52	0.84
Northern Europe	39	48	0.34
Eastern Europe	89	114	0.19
Southern Europe	103	106	0.83
Western Europe	85	95	0.45
Asia	62	42	0.05
Total	428	457	0.33

*EP, extremely preterm (gestational age below 28 weeks)*.

The linear regression analysis showed no significant correlation between the level of lockdown restriction and difference in the number of EP infant admissions (*p* = 0.3; Table 3).

**Table 3:** Number of EP infant admissions in 2020 and 2019 stratified by level of lockdown restriction.

**Level of lockdown restrictions**	**EP infant admissions during the 3 months with the most rigorous lockdown restrictions in 2020 (*n*)**	**EP infant admissions during the corresponding 3 months of 2019 (*n*)**
1 (no difference)	-	-
2	-	-
3	42	37
4	64	66
5 (most severe)	322	354

When investigators were asked to describe the impact of the COVID-19 lockdown on the everyday life of a pregnant woman, 33 out of 46 (72%) answered that they thought the change had been radical or very radical (Table 4). Two out of 46 (4%) answered that they thought the change had been little or very little. Investigators were furthermore asked to classify the likelihood that restrictions outside or inside health institutions in their country/region, could have led to non-admittance of EP infants. Thirty-four out of 46 (74%) answered that they thought it was unlikely or very unlikely (Table 4). Three out of 46 (7%) answered that they thought it was likely or very likely.

**Table 4:** Reporting of exploratory outcome results.

**Likert scale**	**1**	**2**	**3**	**4**	**5**
Impact of the COVID-19 lockdown on the everyday life of a pregnant woman (one being no change, five being radical change)	*n* =0	*n* = 2	*n* = 8	*n* = 19	*n* = 14
Likelihood of restrictions outside or inside health institutions in their country/region could have led to non-admittance of EP infants(one being very unlikely, five being very likely)	*n* = 22	*n* = 12	*n* = 6	*n* = 2	*n* = 1

Of the 46 participating investigators, three reported changes in the structure and organization of perinatal care within their NICU, during the lockdown period. This included reports of nursing staff being rotated to COVID-19 services, increase or decrease in the NICU bed capacity due to structural changes caused by COVID-19 and COVID-19 testing of all patients before admission. One investigator also suspected that online consultations instead of on-site consultations with mothers could have led to a greater amount of intrauterine complications and thus, preterm births.

### *Post-hoc* Analysis Results

The *post-hoc* analysis was based on data from all 46 NICUs. The total amount of NICU admissions during the three most rigorous lockdown months of 2020 was 7,499 and the admissions of same time period in 2019 was 8,362. The overall difference was −10.3 percent. The percentage of extremely preterm infants of total admissions was 5.7 percent in 2020 compared to 5.5 percent in 2019. The difference between regions can be found in Table 5.

**Table 5:** Total NICU admissions in 2020 and 2019 within each pre-defined geographic region.

**Region**	**Total infant admissions during 3 months of most rigorous restrictions in 2020 (*n*)**	**Total infant admissions during the corresponding 3 months of 2019 (*n*)**	**Difference in percent**
North America	1,014	1,154	−12.1
Northern Europe	781	739	+5.7
Eastern Europe	1,297	1,560	−16.9
Southern Europe	1,906	1,951	−2.3
Western Europe	1,472	1,585	−7.1
Asia	1,029	1,373	−25.0
Total	7,499	8,362	−10.3

## Discussion

Based on 885 EP infant admissions from 46 NICUs across 17 countries, this international retrospective, observational study found no major decrease in the number of EP infant admissions during the 3 months with the most rigorous lockdown restrictions during the first phase of the COVID-19 pandemic compared with the corresponding 3 months in 2019. Furthermore, no major changes in the number of EP infant admissions were seen within the geographical regions, although the statistical uncertainty was greater. There was no correlation between levels of lockdown restrictions and changes in the number of EP infant admissions. The *post-hoc* analysis, revealed an overall decrease of 10.3 percent in total NICU admissions, thus the EP infant admission percentage was very constant.

Our study has two important strengths. First, to our knowledge, this study is the largest evaluating the effect of the first phase of the COVID-19 pandemic on EP infant admissions, so far. We surpassed the indicative sample size, which decreases the risk of type II errors, and to some extent compensates for the correction for multiple testing in the secondary outcomes (10). Secondly, the protocol for this study, including the statistical analysis plan, was registered at clinicaltrials.gov before any data was collected, thereby reducing the risk of selective outcome reporting bias (11).

Our study has several weaknesses. First, as COVID-19 is an emergency, we decided to move quickly and therefore, the principal investigators had 3 weeks (August 28th–September 13th, 2020) to report the data. It is plausible that the participation would have been better if we had prolonged the data collection time. Thirty-three NICUs of the SafeBoosC-III consortium did not participate in this study, for reasons which are uncertain. This imposes a potential bias, although it may appear unlikely that this would tend to remove a real effect of the lockdown. Secondly, the data was self-reported, not verified by a third party, and collected in an unblinded fashion, although from well-defined sources. Thirdly, we compared only with the corresponding time period in 2019, not several preceding years, as done in previous studies. The previous studies, however, needed to do that to achieve statistical strength. This was not necessary in our case. It is unlikely that 2019 should have been unrepresentative for the preceding period, due to the large number of participating NICUs. Fourthly, the 3 months where the lockdown restrictions were deemed most rigorous, might not necessarily be the peak months in terms of COVID-19 cases. Thus, the results from this study do not necessarily reflect the number of admissions during the months with most COVID-19 cases. Furthermore, subjective judgements by the local investigators were used to judge the level of lockdown restriction, whether the lockdown had an impact on the life of pregnant women and whether lockdown restrictions could possibly lead to non-admittance of EP infants. This was done to provide simple background information since a more formal data collection for this purpose would have been a major undertaking. The subjective judgements may pose as a limitation in regard to response bias. It is plausible, however, that most of these biases would rather have induced a false association between the change in numbers and the possible explaining factors. The analysis of number of admissions within each geographical region had lower statistical power, due to correction for multiple testing in the secondary outcome analyses, as well as the smaller number of admissions within each region (10).

The most important weakness to consider is the self-selection of the NICUs in the SafeBoosC-III consortium. It is an “*ad-hoc*” group of neonatologists with an academic interest in brain-oriented neonatal care. Therefore, the NICUs that have provided data for this study are typically academic, tertiary NICUs and their admissions of EP infants may not represent EP infants in the regions they serve. It is again, however, difficult to see how this would bias the results as regards the effect of the initial COVID-19 lockdown. Also, only few investigators reported that the COVID-19 epidemic had affected the organization of neonatal intensive care in their regions. Finally, due to the drop in the randomisation rate in the SafeBoosC-III trial, an investigator-bias would most likely to have been in the direction of an exaggerated effect of the lockdown.

In summary, we conclude that our results may be a fair estimate of the general, global effect of the lockdown and that it is likely to have been small, a conclusion that is also supported by results in a previous report (12). Lockdown restrictions causing pregnant women to be more likely to stay home may certainly influence several risk factors for preterm birth, such as stress and prolonged standing at work as well as infections on one side and the clinical processes leading to physician-induced birth on the other side. It is also possible, that in some unfortunate cases, an extremely preterm delivery may have taken the form as a late miscarriage. But our results suggest that these effects have been at most moderate in magnitude.

Despite the number of EP admissions did not significantly differ much in 2020 from 2019, the average number of randomisations in the SafeBoosC-III trial, dropped significantly in March 2020, where the COVID-19 pandemic spread across Europe. Possible explanations for this specific finding, could be that some NICUs participating in the SafeBoosC-III trial, were forced to suspend all clinical research, due to the pandemic. In others, some staff members were re-allocated to clinical duty in COVID-19 departments. Furthermore, some NICUs split the clinical staff into smaller groups, so that fewer staff members were working simultaneously in the NICU, as a preventive measure to avoid the spread of COVID-19 within the department (personal communication). Such measures could all potentially affect the number of randomisations in SafeBoosC-III.

We have studied the immediate effects of lockdown. Delayed effects are also possible. The existence of neonatal networks and national registries will allow stronger epidemiological studies to be done, including examining the effects on the children themselves.

## Conclusion

This larger *ad hoc* study did not confirm previous reports of a major reduction in the number of extremely preterm births during the first phase of the COVID-19 pandemic.

## Data Availability Statement

The original contributions presented in the study are included in the article/Supplementary Material, further inquiries can be directed to the corresponding author.

## Author Contributions

MR, MH, and GG contributed to the conception and design of the study, drafted the protocol, collected data, drafted the manuscript, and gave final approval of the version to be published. TK, ED, AH, SF, MC, CL, IS-K, GN, MW, EP, MF, RR, JT, LCo, LB, GH, PW, EM, JD, RV, DG, ZP, EH, ER-W, HR, AE-K, AMa, SA, SP-B, BK-O, KS, TS, OO, MS, AMe, LA, EE, HF, JS, MB, AP, HO, LCh, GP, MC, and MA contributed to the data collection, revised the manuscript critically for important intellectual content, and gave final approval of the version to be published. All authors contributed to the article and approved the submitted version.

## Conflict of Interest

The authors declare that the research was conducted in the absence of any commercial or financial relationships that could be construed as a potential conflict of interest.
