# Supplementary material for: Extremely Preterm Infant Admissions Within the SafeBoosC-III Consortium During the COVID-19 Lockdown
Source: Front Pediatr. 2021 Jul 12;9:647880. doi: 10.3389/fped.2021.647880 (PMC8310995; doi:10.3389/fped.2021.647880)
Supplement: Supplementary file 1 [file Table_1.DOCX]

**Data sheet**

1. **NICU:**

1. **Author and affiliation:**
2. **Start and end dates for three months of peak (defined as three consecutive months with most rigorous restrictions):**
3. **ELGAN count**

| Count of inf ants GA<28 weeks admitted to your NICU during the peak three months of COVID-19 pandemic | Count of infants GA<28 weeks admitted to your NICU during the same three months in 2019 |
| --- | --- |
|  |  |

1. **Has there been any major changes in the organisation of perinatal care in your area which could be expected to change the number of admissions of extremely preterm infants to your NICU from 2019 to 2020? Tick the box with an x, which you find most correct**

| Yes |  |
| --- | --- |
| No |  |

**If yes, please describe**:

1. **Please describe where/how you have obtained this data (admission logbook, NICU or hospital database, national registry, other: please describe):**
2. **On a scale from 1-5, one being very little change and five being radical change, how would you** **describe the impact of the COVID-19 lockdown on the everyday life of a pregnant woman (tick the box with an x, which you find most correct)**

| 1 |  |
| --- | --- |
| 2 |  |
| 3 |  |
| 4 |  |
| 5 |  |

1. **On a scale from 1-5, one being very unlikely and 5 being almost certainly, how likely do you think that the** **COVID-19 restrictions outside and inside health institutions in your country/region has led to non-admittance of an extremely preterm infant. Causes could be intrauterine death, or no transfer from place of birth (tick the box with an x, which you find most correct)**

| 1 |  |
| --- | --- |
| 2 |  |
| 3 |  |
| 4 |  |
| 5 |  |

1.
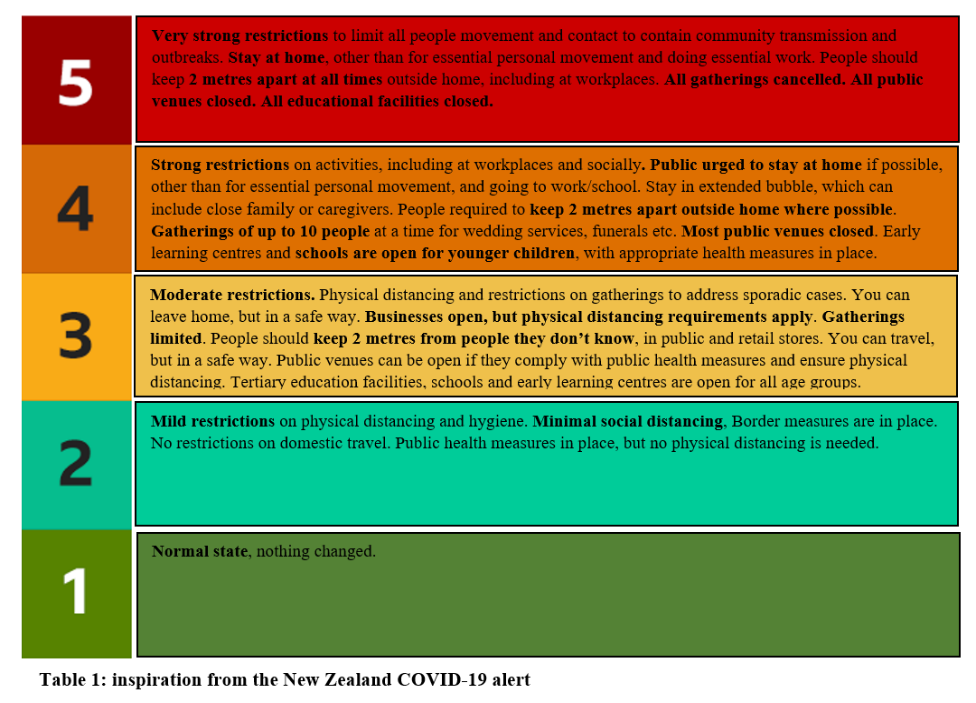
**On a scale from 1-5, one being no change in society and five being very strong change, would you describe the most rigorous restrictions in your country during the three peak months of the COVID-19 pandemic. Please base your answer on the following figure below:**

**Tick the box with an x, which you find most correct:**

| 1 |  |
| --- | --- |
| 2 |  |
| 3 |  |
| 4 |  |
| 5 |  |
